# 790 A Single Institution’s Surgical Model for Pediatric Burns with ≤ 10% Body Surface Area Involvement

**DOI:** 10.1093/jbcr/irae036.331

**Published:** 2024-04-17

**Authors:** Joel Fish, David Lee, Hawwa Chakera, Charis Kelly, Jennifer Zuccaro

**Affiliations:** Hospital for Sick Children, Toronto, ON; Hospital for Sick Children, Toronto, ON; Hospital for Sick Children, Toronto, ON; Hospital for Sick Children, Toronto, ON; Hospital for Sick Children, Toronto, ON

## Abstract

**Introduction:**

Burn injuries continue to be prevalent in the pediatric population and are the fifth most common cause of non-fatal injury according to the World Health Organization. Unlike the adult population, most pediatric burns in the United States are small in size and are often the result of scalds. Despite the high incidence of small burns, a standardized treatment algorithm does not currently exist, and care is often influenced by clinical judgement and resource availability. As a result, various techniques to close the burn wound and promote further healing are currently utilized. This study explores the utility of a multi-stage grafting technique, involving allograft and autograft, for treating small burns (≤ 10% total body surface area (TBSA)) in pediatric patients.

**Methods:**

A retrospective review of patients aged 0-18 years who had a burn that was ≤ 10% TBSA and underwent a multi-stage grafting procedure involving the use of allograft and autograft between 09-01-2018 and 09-01-2022 was conducted. Demographic information including data pertaining to the burn injury was collected for all patients. The primary outcome measure for this study was the percentage of graft take at the graft dressing takedown procedure. All variables were summarized using descriptive statistics. P values < 0.05 indicated statistical significance.

**Results:**

One hundred and sixty-eight patients met the inclusion criteria for this study. The mean time from presentation to allograft surgery was 10.8 days (SD 5.6) followed by autograft surgery approximately one week later. Most patients were discharged within 24 hours following allograft surgery (88.1%) and autograft surgery (80.9%). Mean autograft take was 97.7% (SD 11.1%) with only four patients experiencing significant graft loss requiring subsequent re-grafting. The main causes of graft loss were infection and inadequate excision of the wound bed.

**Conclusions:**

This analysis revealed that patients who underwent a multi-stage grafting procedure involving the use of allograft and autograft experienced minimal graft loss. These positive outcomes demonstrate that the multi-stage grafting technique, which has traditionally been employed for larger burn injuries, can be successfully adapted for smaller burns in children. Moreover, the findings from this study help to address the significant knowledge gap regarding the optimal approach to treating small burn wounds. Further research in this area is warranted to learn more about cosmetic outcomes following multi-stage grafting and determine how it compares to other techniques for treating small burns.

**Applicability of Research to Practice:**

The findings from this study will allow us to begin benchmarking our treatment of small burn wounds with other centers resulting in improvements in patient care and outcomes.

